# Chitosan: A Green Approach to Metallic Nanoparticle/Nanocomposite Synthesis and Applications

**DOI:** 10.3390/polym16182662

**Published:** 2024-09-21

**Authors:** Ilham Ben Amor, Hadia Hemmami, Nedjoud Grara, Omaima Aidat, Asma Ben Amor, Soumeia Zeghoud, Stefano Bellucci

**Affiliations:** 1 Department of Process Engineering and Petrochemical, Faculty of Technology, University of El Oued, El Oued 39000, Algeria; ilhambenamor97@gmail.com (I.B.A.); hemmami.h@gmail.com (H.H.); benamor.asma39@gmail.com (A.B.A.); zsoumeia@gmail.com (S.Z.); 2 Renewable Energy Development Unit in Arid Zones (UDERZA), University of El Oued, El Oued 39000, Algeria; 3 Laboratory of Applied Chemistry and Environment, Faculty of Exact Sciences, University of El Oued, P.O. Box 789, El Oued 39000, Algeria; 4 Department of Biology, Faculty of Nature, Life Sciences, Earth and Universe Sciences, University 8 May 1945, P.O. Box 401, Guelma 24000, Algeria; 5 Laboratoire de Technologie Alimentaire et de Nutrition, Abdelhamid Ibn Badis University, Mostaganem 27000, Algeria; omaima.aidat.etu@univ-mosta.dz; 6 National Institute of Materials Physics, Atomistilor 405 A, 077125 Magurele, Romania; 7 INFN—Laboratori Nazionali di Frascati, Via E. Fermi 54, 00044 Frascati, Italy

**Keywords:** chitosan, metal nanoparticles, stabilization, nanocomposites, water purification

## Abstract

Chitosan, a naturally occurring biopolymer derived from chitin, has emerged as a highly promising instrument for the production and application of metal nanoparticles. The present review delves into the several functions of chitosan in the development and operation of metal nanoparticles, emphasizing its aptitudes as a green reducing agent, shape-directing agent, size-controlling agent, and stabilizer. Chitosan’s special qualities make it easier to manufacture metal nanoparticles and nanocomposites with desired characteristics. Furthermore, there is a lot of promise for chitosan-based nanocomposites in a number of fields, such as metal removal, water purification, and photoacoustic, photothermal, antibacterial, and photodynamic therapies. This thorough analysis highlights the potential application of chitosan in the advancement of nanotechnology and the development of medicinal and environmental solutions.

## 1. Introduction

**Figure 1:**
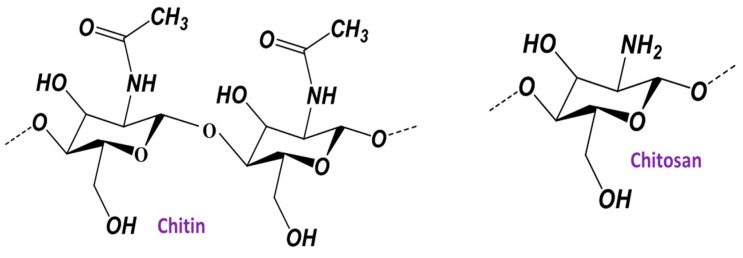
The structure of chitin and chitosan.

The size and shape of metallic nanoparticles are crucial features. The selection of applications for metallic nanoparticles is determined by their size, shape, and the technology used to synthesize them [1]. The optimization of conditions for the controlled production of metallic nanoparticles has long been a subject of interest among nanotechnology specialists. Metallic nanoparticles (MNPs) are highly appealing due to their distinctive characteristics and wide range of applications [2]. The relationship between the shape, size, physicochemical characteristics, and dispersibility of MNPs, and their uses, is well acknowledged. This relationship is influenced by the specific synthesis technique used [3]. Chitosan (CS) is a biopolymer derived from the shells of marine crustaceans [4]. Commercially available chitosan is obtained through the deacetylation process of chitin, a natural biopolymer found in many organisms such as crabs, coral shrimp, mushrooms, lobsters, jellyfish, butterflies, ladybugs, and fungi [5]. N-acetyl glucosamine and D-glucosamine, which are sporadically distributed throughout its composition, sequence, and molecular chain length, make up the positively charged polymer known as chitosan (see Figure 1).

**Figure 2:**
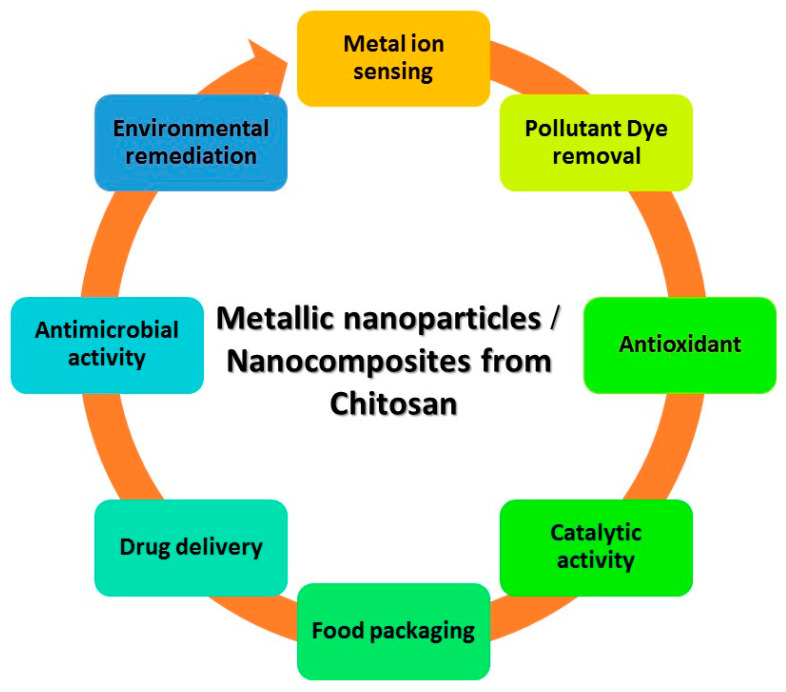
Applications of metallic nanoparticles based on chitosan.

The acetylation level of chitosan is determined by the molar fraction of N-acetylated units (DAs) or as a degree of deacetylation (DD%), which represents the proportion of deacetylated units. Various techniques, such as chemical extraction (including chemical demineralization and chemical deproteinization), enzymatic deproteinization, biological extraction, fermentation, and chemical deacetylation, have been employed to convert chitin into chitosan [6]. Chitosan facilitates electrostatic attraction with biopolymers that have a negative charge and contact with cellular membranes. CS has been produced in many forms, such as films, foams, fibers, hydrogels, and nanoparticles. CS exhibits inter- and intra-molecular hydrogen bonding due to the presence of amine and hydroxyl groups. Thus, it possesses a firm and well-defined crystalline arrangement [7]. The addition of metallic nanoparticles to CS enhances its potential for widespread application. The applications of these materials encompass a wide range of fields, such as nonlinear optics, catalysis, adsorption, heavy metal ion sensing, antimicrobial activity, environmental remediation, catalytic activity, dye removal, antioxidant properties, drug delivery, bioimaging, wound healing, sensing, and anti-cancer activity (see Figure 2) [8].

## 2. Properties of Chitosan

Second only to cellulose, chitin, the precursor to CS, is the most often occurring biopolymer in nature. It is quite common in many organisms including fungi, crustaceans, and insects. Comprising N-acetyl-D-glucosamine units, chitin is a biopolymer. When deacetylated, it produces a polymer consisting mostly of β-1,4-D-glucosamine units. This polymer is called chitosan (see Figure 1) [9]. It has gained considerable interest because of its distinct characteristics and extensive array of applications. Below are some fundamental characteristics of CS.

### 2.1. Degree of Deacetylation (DD)

DD is determined by the ratio of glucosamine groups to the total amount of glucosamine and acetylglucosamine groups. The DD value of a polymer serves as a distinguishing factor between CS and chitin. A polymer is classified as chitosan if its DD value exceeds 60%. In order to determine DD, several analytical approaches are employed, including infrared spectroscopy, near-infrared spectroscopy, UV spectroscopy, potentiometric titration, and magnetic resonance [10].

The extent of deacetylation greatly affects the solubility and solution characteristics of CS. Mw and DD of chitosan have an impact on its physical, chemical, and biological properties. Various experiments have been conducted using many chitosan samples, all of which had similar Mw but varying levels of disulfide bonding DD, ranging from 70% to 95%. It was shown that these factors are correlated with the physicochemical properties of the material. CS samples with high DD exhibit a higher abundance of crystalline regions compared to those with low DD. Higher DD levels lead to increased elasticity and tensile strength [11].

### 2.2. Molecular Weight (Mw)

The Mw of CS varies according to its source and the conditions of deacetylation, including temperature, time, and base concentration. The degradation of chitosan and a subsequent decrease in its molecular weight occurs in the presence of dissolved oxygen in the solution media. Additionally, elevated temperatures (≥280 °C) disrupt the polymer chains in chitosan and reduce its Mw. However, it has been shown that the crystallinity, DD, and Mw of these materials affect how effective they are. Temperature, reagent concentrations, alkaline step repetition, duration, and ambient conditions during the deacetylation all significantly affect the Mw and DD of chitosan [6]. Enzymatic and chemical techniques are used to manufacture chitosan [12]. Poor structural order (glucose ring) and low product yields are just two of the disadvantages of the chemical process. Additionally, the chemical process’s use of acids and alkalis may have an adverse effect on the environment [12]. An alternate, more environmentally friendly, method of producing chitosan is to use the enzymatic process. Instead of using acidic treatment, lactic acid-producing bacteria have been employed throughout the enzymatic process to demineralize crustacean shells. The resulting calcium lactate may be precipitated and eliminated after the calcium carbonate and lactic acid reaction. Proteases from bacteria (*Serratiamarcescens*, *Bacillus subtilis*, and *Pseudomonas aeruginosa*) are employed to deproteinize crustacean shells. *Bacillus* sp. and *Serratia* sp. are bacteria that may be utilized to make chitosan since they also produce chitin deacetylase. The efficient deacetylationis accomplished by sometimes rinsing the intermediate byproduct using water through the alkaline treatment. CS has a B500 kDa average Mw and 100% DD. When treating alkaline during the first hours (50% NaOH) at 100 °C, the DD rises quickly to around 68% and then gradually rises over time [13].

Chitosan with a low Mw (LMWC; 50 kDa), chitosan with an average Mw (50–250 kDa), and chitosan with a big Mw (HMWC; >250 kDa) are the three main forms of chitosan that are categorized based on Mw. According to some publications, LMWC has improved antibacterial, antifungal, intestinal disaccharidase, lipid metabolism, anticancer, and mucoadhesive characteristics [14]. In addition, the Mw is crucial for the rheological characteristics of the biopolymer. It has an immediate effect on the creation of chitosan-based biomaterials [14]. LMWC is used as an anticreasing agent to offer a finishing agent when treating cotton materials for anticreasing. HMWC is used in quaternized chitosan films due to its free radical scavenging and water solubility properties. It is also used in high-performance cells and affects polymer aggregation and phase separation.

### 2.3. Solubility

The process of deacetylation converts certain N-acetylglucosamine components of chitin into glucosamine units, resulting in the formation of CS. CS, with a pKa of around 6.5, exhibits solubility in acidic aqueous environments because of the abundance of protonated -NH_2_ groups in its molecular structure. CS becomes soluble when about 50% of its amino groups are protonated. Modifying factors such as polymer Mw, pH, DD, temperature, and polymer crystallinity might potentially impact the solubility of CS [9].

The process of homogeneous deacetylation of chitin by alkali treatment enables the creation of polymers that may dissolve in aqueous acetic acid solutions, even with a DD of as low as 28%. On the other hand, heterogeneous deacetylation, which involves alkali treatment at high temperatures, never achieves this level of deacetylation. In addition, the samples are capable of dissolving in water, with a degree of dissolution of 49%. This phenomenon can be attributed to the modification of the polymer’s crystalline structure and the subsequent rise in the number of glucosamine units following a uniform deacetylation process. The degree to which crystal size and perfection are reduced or a new crystal structure resembling β-chitin is present is determined by the polymer’s DD [9].

### 2.4. Viscosity

The viscosity of polymers is an important technical characteristic due to the difficulty in controlling very viscous solutions. Furthermore, viscometry is a reliable tool for determining the Mw of chitosan due to its simplicity and efficiency. However, it is not definitive and thus necessitates the determination of specific constants for the solvent used [15].

The viscosity increases with an increase in DD. Chitosan, when dissolved in water, exhibits several forms depending on the extent of deacetylation. Deacetylated chitosan exhibits a less compact structure and more flexible chains due to the repulsion between charges. CS with low DD exhibits either a rod-like or coil-shaped structure due to the low charge density in its polymer chains. The viscosity of chitosan varies based on its concentration and temperature. The viscosity of chitosan rises as the concentration of the medium is higher or when the temperature is lower. The Mw of chitosan is highly associated with its viscosity. CS with a high Mw exhibits a higher viscosity compared to chitosan with a low molecular weight. Viscosity is affected by both physical and chemical processes. Viscosity can be decreased using methods such as prolonging the grinding duration, applying heat, autoclaving, using ultrasound, and employing ozonation [15].

### 2.5. Biocompatibility and Thermal Stability

CS has been found to be highly biodegradable, mostly due to the ability of lysozyme and chitinase to break down its molecular chains under normal physiological conditions. Chitosan’s biodegradability makes it advantageous for many applications like drug administration, tissue engineering, insecticides, and other medical and agricultural uses. The polymer degradation is indicated by an exothermic peak observed within the temperature range of from 279.45 to 281.89 °C. Based on the literature, CS undergoes degradation at a temperature of around 250 °C. The exothermic process is caused by the crystallization of chitosan [16].

### 2.6. Relationship between Degree of Deacetylation, Molecular Weight, and Nanoparticles

Greater Mw often results in higher viscosity, which can impact the production of nanoparticles, leading to bigger particle sizes. This is due to the fact that polymers with greater Mw have a tendency to create longer and more intertwined chains, which in turn makes it more challenging to break them down into smaller nanoparticles during the manufacturing process. Smaller and more uniform nanoparticles are commonly linked to polymers with lower molecular weight due to the fact that their shorter chains can be processed more readily into small, well-defined particles [17].

Elevating the DD results in a higher quantity of unbound amino groups (NH_2_) throughout the polymer chain. The amino groups in chitosan become positively charged in acidic circumstances, which improves its solubility in water and strengthens its interaction with negatively charged molecules. This can result in improved interaction with anionic medicines or molecules during the formation of nanoparticles, hence increasing the drug-loading capacity and strengthening the stability of the nanoparticles. Reducing the DD leads to a decrease in the number of amino groups and an increase in the number of acetylated units, resulting in a decrease in the solubility and reactivity of the polymer. This can result in the creation of bigger nanoparticles that are less stable and have a reduced effectiveness in encapsulating drugs [17].

Increased Mws of polymers typically result in the formation of bigger nanoparticles, mostly because of heightened viscosity and chain tangling. In contrast, polymers with lower molecular weight have a tendency to create smaller nanoparticles due to the fact that their shorter chains are easier to handle [18]. An enhanced DD enhances the stability of nanoparticles by strengthening the electrostatic contacts between negatively charged molecules and the positively charged amino groups. Additionally, this can improve the drug loading efficiency and release profile of the nanoparticles [19].

Mw and DD synergistically impact the overall characteristics of nanoparticles. For instance, a polymer with a high molecular weight and a high DD might result in the formation of bigger and more stable nanoparticles that possess excellent qualities for encapsulating drugs. On the other hand, a polymer with a low Mw and a lesser DD might produce smaller, less stable nanoparticles that may have a poorer effectiveness in loading drugs [20]. An in-depth comprehension of the correlation among Mw, DD, and nanoparticles is essential for the development of polymer-based nanoparticles tailored for specific purposes, like drug delivery. The dimensions, stability, and release characteristics of the nanoparticles are crucial in determining their effectiveness. These factors are meticulously controlled through careful molecular weight selection and precise regulation of DD [21].

## 3. Chitosan’s Effects on Metal Nanoparticle Formation and Functionalization

**Figure 3:**
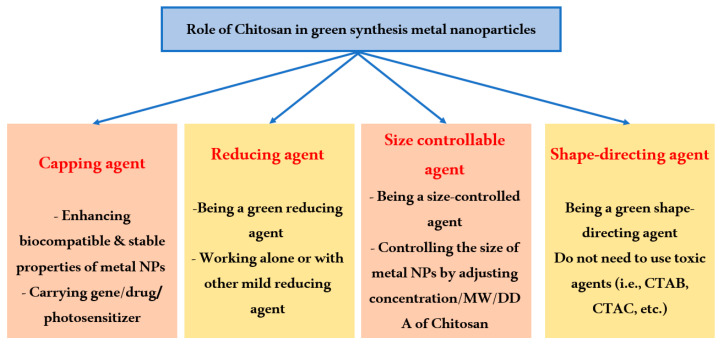
An overview of chitosan’s role in environmentally friendly metal nanoparticle production.

CS can impact both the creation and modification processes of metal nanoparticles (see Figure 3) [22]. Upon the addition of the cationic polymer CS to the reaction solution, electrostatic interaction occurs between the positively charged CS and the negatively charged NPs, which are coated with a negative capping agent [22]. Alternatively, the interaction happens when chitosan is absorbed onto the surface of NPs, leading to the formation of a chitosan shell around the nanoparticles [23]. Regarding the formation process, chitosan can be incorporated either before or during the production of the nanoparticles. Chitosan can serve as a reducing agent, stabilizing agent, shape-directing agent, and size-controlling agent in the synthesis of NPs. In the functionalization process, CS is employed to modify the surface of metal nanoparticles to enhance their biocompatibility and drug-carrying capabilities.

### 3.1. Chitosan as a Stabilizer

**Figure 4:**
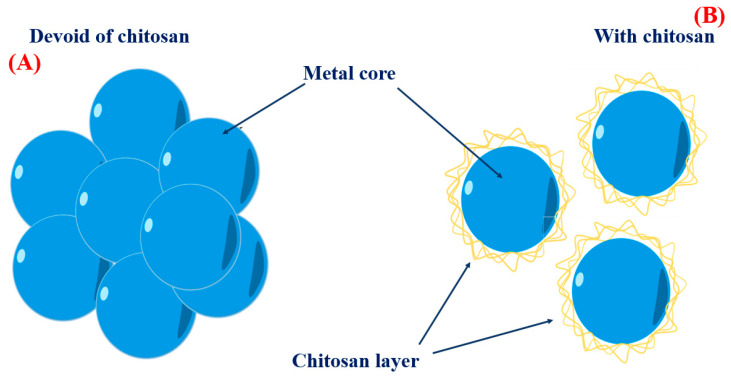
Metal nanoparticle dispersion and the influence of CS capping. (**A**) The metal nanoparticles’ aggregation in solution in the absence of a CS capping agent. (**B**) The NPs are evenly distributed and in solution with a CS capping agent.

When creating metal nanoparticles (NPs), natural polymers are frequently chosen to act as stabilizers. Due to its biocompatibility, availability, and highly positive charge, chitosan is an effective stabilizer for metal nanoparticles. Figure 4A demonstrates that bare metal nanoparticles tend to form clusters in solution because of the attractive forces known as Van der Waals interactions between the untreated metal surfaces. On the other hand, CS acts as a physical barrier with a high concentration of positive charges surrounding the metal. The cohesive force resulting from the strong electrostatic connection between positively charged metal nanoparticles facilitates the creation of uniform solutions containing metal nanoparticles, as depicted in Figure 4B. Several investigations have proven the favorable efficacy of CS in stabilizing metal nanoparticles. For instance, CS was employed as the stabilizer in the synthesis of AgNPs using an environmentally friendly method involving an electrochemical oxidation/complexation procedure with UV irradiation reduction [24]. Gold nanoparticles (AuNPs) were produced using citric acid and CS as stabilizers. The AuNPs produced in this study exhibited stability in the aqueous phase, showing no signs of agglomeration [25]. Biomedical applications like gene/drug delivery, tissue engineering, and photo-based therapy have made substantial use of chitosan oligosaccharide (COS), a soluble polymer produced from CS, to coat metal nanoparticles [26]. COS was employed as a green reducing agent and stabilizer in a one-step process to synthesize AuNPs for gene transfer purposes [27]. The presence of positively charged amino groups in COS enhanced its binding affinity with plasmid DNA.

Manikandan et al. [28] illustrate the production of CuNPs by the introduction of an acidic chitosan solution into a CuSO_4_ solution. Chitosan facilitates the stable production of nanoparticles. The existence of a peak at 536 nm in the UV–Vis spectroscopy suggests the reduction of Cu^2+^ ions in the presence of chitosan, resulting in the creation of extremely monodisperse nanoparticles. Upon the addition of copper sulfate to the chitosan solution, the Cu^2+^ ions would bind to CS macromolecules using electrostatic interactions. The oxygen atoms in the hydroxyl and ether groups of CS, which have an abundance of electrons, are prone to interacting with metal cations that have a positive charge.

Qian et al. [29] illustrate the utilization of chitosan for the production of stable nanoparticles through the process of chelating Fe^2+^ and Fe^3+^ ions. The chitosan nanoparticles operate as a framework that contains iron ions, which leads to the improved antibacterial effectiveness of the resultant Fe-loaded chitosan nanoparticles.

### 3.2. Chitosan as an Environmentally Friendly Reducing Agent

It is a normal practice to utilize toxic reducing agents that release environmentally hazardous substances when making nanoparticles chemically. The direct use of chemically generated nanoparticles for biological purposes was hindered by the presence of toxic capping, which is difficult to separate from the nanoparticles. If nanoparticles are to be used in biomedicine and environmental protection, they must be made with eco-friendly methods and materials. Numerous studies have shown that during their green manufacturing, copper nanoparticles (CuNPs) [28], gold nanoparticles (AuNPs) [25,30], and silver nanoparticles (AgNPs) [30,31] may all be reduced and stabilized using CS. Carapeto et al. [32] attempted to put together the Ag ion’s CS reaction mechanism using X-ray photoelectron spectrum and UV/Vis absorption spectroscopy. The experimental results showed that Ag reduction in chitosan aqueous solutions begins early on, even at ambient temperature, and that the reaction accelerates with increasing temperature. The free electron necessary to convert Ag^+^ to Ag^0^ and form carbonyl groups is supplied by the oxidation of alcohol or the glucosidic groups of several functional groups in CS. Through UV–Vis spectra and X-ray photoelectron spectroscopy (XPS), which revealed an absorption peak at around 262 nm, corresponding to the π* ← n transition in a carbonyl group, scientists confirmed that carbonyl groups are the primary products in the reaction medium. Furthermore, coating or encapsulating metal with cationic chitosan results in the production of positively charged nanoparticles and long-term stability in terms of aggregation [33].

Wongpreecha et al. [34] offered an alternative elucidation of the response mechanism of CS. The green process was conducted in an autoclave at a high temperature of 120 °C and a high pressure of 15 psi. Ag^+^ ions formed coordination bonds with the electron pairs of nitrogen and/or oxygen atoms on the chitosan backbone in an acidic environment with a pH of 4, which is lower than the pKa value of CS. Subsequently, the Ag^+^ ions underwent reduction to Ag^0^ through the interaction with a lone pair electron of oxygen in CS at elevated pressure and temperature. CS functioned as a sterically and electrostatically stabilizing agent for the nanoparticles that were produced. Utilizing a core-shell nanostructure is a highly successful approach to improving the performance of NPs. Chitosan was utilized as a green reducing agent to create nanoparticles with a core-shell nanostructure for use in the field of biomedicine. Wang et al. [35] synthesized a core-shell nanocomposite called Cu@Pd-chitosan using the green technique and natural chitosan. The Cu@Pd–CS composite exhibited excellent stability, sensitivity, and resistance to interference.

The work conducted by Masood et al. [36] outlines a technique for producing magnesium oxide nanoparticles (MgONPs) using a green chemical approach. The process involves utilizing chitosan polymer as a reducing agent. The FTIR spectra verified the existence of many crucial functional groups in the produced MgONPs. Furthermore, the presence of a peak at 358 nm in the UV–Vis spectrum confirmed the creation of nanoparticles. The XRD pattern indicated that the nanoparticles had an average size ranging from 29.4 to 34.7.

### 3.3. Chitosan as a Size-Controlling Agent

**Figure 5:**
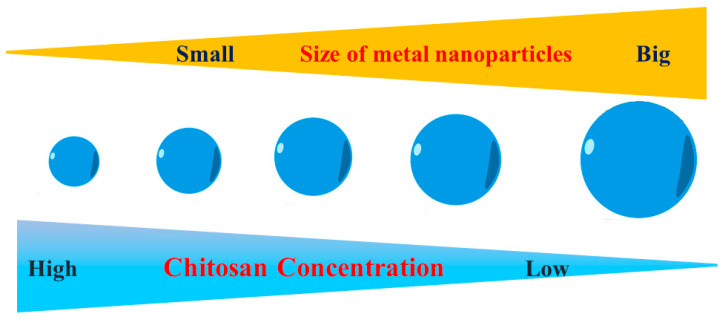
The relationship between metal nanoparticle size and chitosan content.

CS also plays a role in controlling the size of NPs throughout the green synthesis process. Based on the results from the UV–Vis absorption spectrum, Kalaivani et al. [37] found that the synthesis of AgNPs was significantly enhanced in the presence of CS. Furthermore, the size of AgNPs exhibited a significant reduction when the concentration of chitosan was increased (Figure 5). Our recent studies have once again validated this finding. The size of metal nanoparticles, specifically PdNPs [38] and AuNPs [39], decreased as the concentration of CS was raised. We have proposed a theory to elucidate the impact of CS on the dimensions of the nanoparticles produced. When metal nanoparticles are formed in the presence of chitosan, the positively charged CS forms a powerful electrostatic contact with the metal nuclei. Increased CS concentration results in enhanced chitosan–metal nuclei interaction. This robust contact hinders the attachment of precursors to the metal nuclei. Therefore, metal nuclei cannot undergo further growth when exposed to a high concentration of chitosan solution.

Mona et al. [40] primarily investigates the production of CS/tripolyphosphate nanoparticles. The main focus is on effectively managing the size and polydispersity index of these nanoparticles in various microchannels. CS serves to impact the size and consistency of the nanoparticles produced, as assessed using computational fluid dynamics modeling and experimental verification. The work by Ilham et al. [41] utilizes CS as a size controlling agent, which plays a vital role in regulating the size, shape, and characteristics of the ZnO nanoparticles (ZnONPs), including their optical bandgap and photocatalytic efficacy. The use of various chitosan sources, such as crab shells, shrimp shells, and *Streptomyces griseus* bacteria, has an impact on the dimensions and antibacterial effectiveness of ZnONPs. This suggests that CS plays a crucial role in defining the size and associated characteristics of the nanoparticles. The study shows that chitosan affects the size of ZnONPs crystallites, which can range from 20 to 80 nm depending on the source of CS.

### 3.4. Chitosan as a Shape Orientation Agent

While employing the chemical approach, toxic capping agents such as trisodium citrate, CTAB, and CTAC are commonly used as shape-directing agents for metal nanoparticles [42,43]. For sustainable biomedical uses, replacing metal nanoparticles with natural molecules can improve their biocompatibility. As a structure-directing agent, CS was used to electrodeposited AgNPs on disposable pencil graphite electrodes [44]. According to the scientists, AgNPs display unique morphologies when CS is present. AgNPs, on the other hand, are irregularly shaped and lack CS. By changing the parameters of the experiment, AgNPs have been created in a range of morphologies, such as hexahedron, leaf, and dendritic [44].

Moreover, CS can be changed to enhance its capacity to regulate the shape of nanoparticles. Utilizing cationic N-trimethylamine groups, anionic ligands, and o-carboxymethyl are a few examples of these changes. The generation of various AuNPs forms is regulated by the positive and negative charges on the CS. As per Reference [45], gold nanochains, nanoneedles, and nanoflowers can be fabricated using thiolate-functionalized carbon starch as a soft template. The thiol group of the CS can engage in significant interaction with AuNPs that belong to distinct assemblies. Moreover, it has been shown that the architecture of a gold nanocrystal can be self-assembled utilizing a peptide that has an aromatic component [46].

Gallic acid (GA) and folic acid (FA) -N-trimethyl CS (FA-GA-TMC) were shown to be effective in promoting the self-assembly of SeNPs with a cubic form in another study [47]. Three crucial structural characteristics can be acquired by modifying CS as follows: (1) the quaternized CS’s N^+^(CH_3_)_3_ group’s positive charge improves the electrostatic interaction between the stabilizer’s positive charge and the negatively charged surface of SeNPs [48]; (2) the hydrophobic components of FA and GA contribute to a π-π stacking interaction, which serves as a rigid template; and (3) the hydrogen bonding groups from the FA, GA, and chitosan backbones form a complementary trio. The big hydrophobic groups of the GA and FA are presented externally as a result of the interaction between the hydrophilic group of N^+^(CH_3_)_3_ and the surface charge of the SeNPs. This facilitates the hydrogen-bonding and π-π stacking interactions between neighboring particles and further opens the way for the assembly into cubic-like SeNPs [48].

### 3.5. Chitosan as a Multifunctional Tool for Metal Nanoparticle Preparation

**Figure 6:**
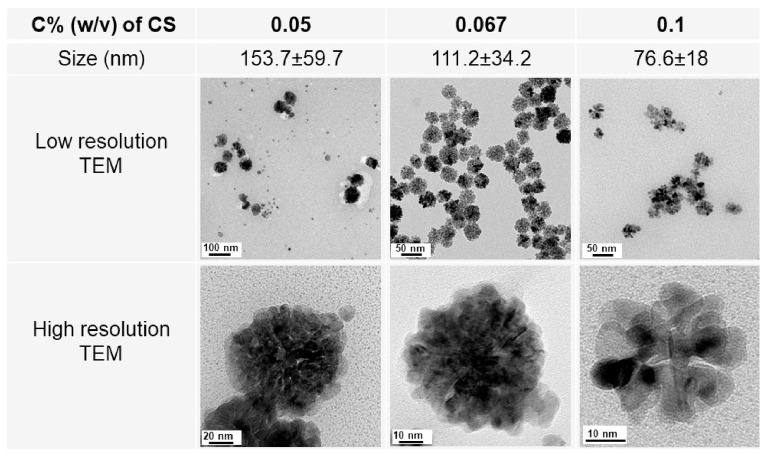
TEM and size of PdNPs synthesized with various concentrations of CS.

According to recent reports, chitosan can serve as a versatile agent in the synthesis of metal NPs, including ZnONPs and palladium nanoparticles PdNPs [41]. To synthesize porous flower-shaped palladium nanoparticles (see Figure 6), we established a revolutionary green technique in 2019 [38]. In this method, CS serves as a multifunctional agent that includes a stabilizer, form director, and size controller. We used different concentrations of CS to set up the studies, and vitamin C, a green reducing agent, was utilized to convert Pd^III^ to Pd^0^. Despite not using a form-directing agent, we were able to obtain PdNPs with the flower shape in every trial. In this case, CS was crucial to the development of PdNPs with a flower-like morphology. Following their synthesis under the effect of vitamin C, chitosan strongly interacted with Pd nuclei to coat the surface of the PdNPs. PdNPs with a porous flower shape were produced when Pd nuclei continued to grow in an anisotropic direction in the presence of chitosan. The CS layer’s development hampered the reduction process on the Pd nuclei’s surface. PdNPs were entirely mature at this point. We found that smaller PdNPs were produced when the CS concentration was increased. A greater concentration of CS in the solution will cause the CS layer to develop more quickly and result in smaller PdNPs. This idea allows for the creation of PdNPs with the appropriate size by varying the quantity of additional CS applied. In the other work [39], we created gold nano-stars (AuNSs) in an environmentally friendly manner by using chitosan as a multifunctional agent. Strong interactions with Au nuclei allow CS to connect to the core of AuNPs because of its strongly positive charges in acidic conditions. The anisotropic growth of gold nanostructures has been facilitated by the tip’s strong growth at the loose contact areas between the core and CS, resulting in a shape resembling a star. Ag nuclei and CS interact less strongly in an environment with a higher pH, making it impossible for CS to regulate the growth of Ag nuclei.

## 4. Preparation of Nanocomposites—Metallic Nanoparticles from CS

CS-based metallic nanocomposites have been made using several techniques for a range of metal nanoparticles, including Cu, Fe, Ag, Au, Zn, Ti, V, Ni, Cr, Co, and others (see Table 1). Several significant techniques for the manufacture of metallic nanoparticles using the biopolymer CS include co-precipitation, green synthesis, in situ precipitation, ex situ precipitation, and the hydrothermal approach. Using the in situ method, nanoparticles are produced directly in the chitosan matrix, where CS serves as a capping and reducing agent [49]. To increase the material’s mechanical strength or thermal stability, the metallic ions are first added to the chitosan solution, where they are reduced to form nanoparticles and essentially embedded into the polymer matrix [50].

Better nanoparticle integration, higher stability, and less chemical usage are just a few benefits of this method, which also makes it more environmentally friendly [51]. The performance of the material may be impacted by the in situ method’s limitations on the number of nanoparticles incorporated in the matrix and its potential to produce problems with nanoparticle size and distribution control [52].

Conversely, the ex situ technique entails the individual synthesis of NPs prior to their incorporation into the chitosan matrix [53]. This approach forms the nanocomposite by first preparing the nanoparticles by methods including chemical reduction, green synthesis, or hydrothermal processes, and then combining them with the chitosan solution [54]. This method allows for more control over the size and form of the NPs, as well as the ability to modify their surface prior to integration and to achieve higher nanoparticle loading [55]. However, the synthesis is frequently more time-consuming and necessitates the use of extra stabilizing agents, which might result in nanoparticle aggregation during the integration process. Each strategy has distinct advantages and disadvantages, and the choice between them relies on how the nanocomposite is going to be used [56].

CS has a dual role as both a capping and reducing agent in the production of metallic NPs. Chitosan acts as a cation and forms complexes with anions to create nanocomposites and nanoparticles. Au nanoparticles stabilized with CS were synthesized without the use of a reducing agent [57]. The research on the immobilization of AuNPs and AgNPs within CS conducted by Sanpui et al. [58] examined the utilization of a green synthesis method for producing AgNPs and AuNPs. The size and stability of metallic nanoparticles are influenced by both the molecular weight and concentration of CS. The researchers in Lupusoru et al.’s [5] study synthesized and examined CS-coated AuNPs to determine the stability of AuNPs of various sizes in relation to CS molecular weight and concentration.

AuNPs were synthesized using CS as a stabilizer and thiamine pyrophosphate (TPP) as a catalyst to investigate the impact of varying concentrations of chitosan on the size and morphology of AuNPs without the use of any other reducing agents. CS-capped AuNPs have been utilized for the detection of heavy metal ions by monitoring variations in surface plasmon resonance (SPR). The research conducted using Sanpui et al. [58] discovered that Au–CS nanocomposites are well-suited for selective electrochemical sensors, particularly for the detection of antioxidants and the calculation of the polyphenol index in wines. Copper–chitosan NPs were synthesized using an eco-friendly approach, and their antibacterial activity was then investigated [28]. Preparations were made for immobilizing cellulase using a support made of magnetic CS nanoparticles. According to Zang et al. [59], the immobilized cellulase maintained 50% of its original activity after undergoing 10 cycles. The preparation of CS–titanium oxide fibers and zero-valent NPs was carried out using the hydrothermal approach, as described by Ali et al. [60]. Chaudhary et al. [61] produced zinc-encapsulated CSNPs to enhance the productivity of maize crops. The in situ process is widely recognized as the most renowned and straightforward approach for creating metallic nanocomposites based on chitosan. The synthesis of a cellulose filter paper covered with a nickel–CS nanocomposite was achieved utilizing the in situ approach [62]. Chromium-loaded chitosan nanocomposites were manufactured using an in situ method [63]. Table 1 displays several techniques and metallic nanocomposites that have been made utilizing CS as a biopolymer for capping and complexing purposes.

**Table 1:** Various techniques for creating metallic nanoparticles and nanocomposites based on chitosan.

Ref.	Size (nm)	Characterization	Metal
[28]	20–30	UV–Vis, FTIR, TEM, EDS, and XRD	Copper–Chitosan Nanoparticle
[64]	25	UV–Vis, EDX, STEM, and XRD	Chitosan-Stabilized Copper Nanoparticles
[65]	10	TEM, SEM, X XRD, and UV–Vis, infrared and X-ray photoelectron spectroscopies	CS–AgNPs
[66]	3.5–6.0	XRD, FE-SEM, UV–Vis, EDAX, FTIR, and TEM	Chitosan–copper oxide nanocomposite
[66]	CuO NPs (29.07 nm) and MgO NPs (14.55 nm)	UV–Vis, FTIR, and XRD	Chitosan–CuO-MgO Polymer Nanocomposites
[67]	10–25	SEM, XRD, and FTIR	Chitosan–CuO bio-nanocomposite
[29]	195.2	Zeta potential	Fe-loaded chitosan nanoparticles
[68]	/	UV–Vis and TEM	AuNPs
[37]	10 ± 60	UV–Vis, FTIR, TEM, XRD, and AFM	AgNPs
[69]	10	UV–Vis, EDS, XRD, DLS, FTIR, XPS, and TEM	CS–AuNPs
[70]	75.97	SEM, XRD, VSM, and FTIR	Fe_3_O_4_–CuO–Chitosan Nanocomposites
[71]	5–10	SEM, UV–Vis, FTIR, TEM, and XRD	Chitosan–Zinc Oxide Nanoparticles
[72]	20	UV–Vis spectroscopy, XRD, FFT-IR, TGA, DSC, FE-SEM, EDX, AFM, HR-TEM, XPS, and zeta potential analyser	Chitosan–silver nanocomposite
[73]	6–11	FTIR, FESEM, and EDX	Chitosan–MgO nanocomposite
[74]	15–20	FTIR, SEM, and XRD	Chitosan–MgO nanocomposite
[75]	44.80	XRD, FTIR, EDS, TEM, and FESEM	Chitosan–AgNPs
[76]	-	TEM, FTIR, UV–Vis, and TEM	AgNPs
[76]	17	SEM, XRD, and FT-IR	Chi–CuO
[40]	124.3	/	Chitosan–tripolyphosphate nanoparticles
[77]	20	UV–Vis, FTIR, XRD, AFM, and TEM	CS–Ag nanocomposites
[78]	6 to 18	FTIR, XRD, SEM, and TEM	Chitosan–silver nanocomposites
[79]	80	SEM, Zeta potential, and XRD	AgNPs
[80]	2.1 ± 0.3	FTIR, UV–vis, Zeta potentials, TEM, XPS, and XRD	PtNPs
[81]	130	FTIR, XRD, and FESEM	Chitosan–ZnO nanoparticles
[82]	22	XRD, Zeta potential, TEM, and TGA	Chitosan–zinc oxide Nanocomposites
[83]	40	SEM	ZnO–chitosan Nanoparticles
[84]	-	FT-IR, XRD, SEM, and WDX	Chitosan–zinc oxide nanoparticle
[85]	58	FT-IR, XRD, and SEM	Chitosan–ZnO nanoparticles
[86]	55	FT-IR, SEM, and WCA	Chitosan–zinc oxide (ZnO) nanocomposite
[87]	MgO NPs: 17 nm, and ZnO NPs: 29 nm	DRX, FT-IR, UV–vis, and SEM	ZnO NPs and MgO NPs
[88]	3–8	XRD, FTIR, and TEM	Chitosan–Ag nanoparticle
[60]	26.51	FE-SEM, EDX, XRD and FTIR	Chitosan–TiO_2_
[27]	3–15	UV–vis, FT-IR, and TEM	Chitosan–gold nanoparticle
[89]	60	XRD, FE-SEM, UV–DRS, and XPES	ZnO
[41]	20–80	DRX, FT-IR, UV–vis, and SEM	ZnO NPs
[90]	34.5	XPS XRD, FTIR, TGA, and TEM	Au NPs
[91]	-	XRD, BET, FTIR, and SEM	Chitosan–Fe_2_O_3_nano composite
[92]	-	DRX, FT-IR, TEM, and TGA	Chitosan–zinc oxide hybrid composite
[93]	20	TEM and SEM-EDX	CuO–chitosan nanocomposite
[94]	17.8	UV–vis, FT-IR, and TEM	CS–AuNPs

## 5. Factors Affecting the Synthesis to Control the Properties of Nanoparticles

The synthesis factors, such as pH, CS concentration, temperature, ionic strength, and the inclusion of cross-linking agents, have a substantial impact on the characteristics of CS-modified nanoparticles. Comprehending and enhancing these factors is essential for customizing the dimensions, form, and durability of the nanoparticles; the following are the most important influencing factors.

### 5.1. pH

The pH of the reaction medium is a crucial determinant in the production of nanoparticles modified with CS. CS exhibits solubility in acidic environments, but it undergoes a transition to insolubility when the pH level rises. CS undergoes protonation and establishes robust electrostatic connections with metal ions, particularly Ag^+^, at acidic pH values (about 4). This contact aids in the stabilization of nanoparticles and inhibits their aggregation. This interaction results in the formation of nanoparticles that are evenly distributed and have consistent sizes. On the other hand, elevated pH levels might disturb these interactions, leading to nanoparticles with different shapes or sizes. Consequently, it is crucial to optimize the pH in order to obtain the intended characteristics of the nanoparticles. According to Hashem et al. [95], the stabilizing agents significantly diminish the silver ions at a pH of around 4, at which point the CS/AuNPs are smaller and more evenly distributed, with an average size of 15–20 nm. However, the reduction process is less regulated at a higher pH (around 7), leading to bigger and more polydisperse CS/AuNPs, with diameters ranging from 30 to 40 nm. Higher pH values also cause the CS/AuNPs to become less stable and aggregate more. According to Yuhang et al. [96], pH is important for the development and stability of CS/TPP nanoparticles, with a pH of about 4.0 being associated with the best stability. Because chitosan is protonated at this pH, it interacts with TPP well to improve the synthesis and stability of CS/TPP. Particle aggregation and decreased biomolecular absorption efficiency result from chitosan’s deprotonation, which decreases its interaction with TPP and makes the particles less stable at pH 6.0.

### 5.2. Chitosan Concentration

The concentration of CS in the mixture being reacted influences the degree to which chitosan is adsorbed or linked to the nanoparticles. Increased quantities of CS often result in thicker coats on the nanoparticles. An augmentation in the thickness of the coating can improve the durability of the nanoparticles, but it may also have an impact on their size. Research has demonstrated that increasing the concentration of CS often leads to the formation of smaller nanoparticles. This is because the biopolymer’s powerful stabilizing properties restrict the expansion of metal nuclei. Therefore, the concentration should be modified according to the desired attributes of the resulting nanoparticles. A study by Chitradurga et al. [97] examined the influences of increasing CS concentrations from 0.1% to 1.0% (*w*/*v*) and discovered that larger CS concentrations produced smaller and more stable nanoparticles. In particular, AgNPs averaged 10–15 nm in size at 1.0% chitosan concentration, but they were 20–30 nm at a 0.1% chitosan concentration. Furthermore, the stability of the nanoparticles was enhanced by increasing CS content, which led to greater dispersion and less aggregation. This work shows that regulating the chitosan content is essential to maintaining the stability, size, and form of AgNPs for a range of applications.

### 5.3. Temperature

The temperature has a substantial impact on the kinetics of CS adsorption and coupling [98]. Higher temperatures expedite the process of metal ion reduction and the subsequent creation of nanoparticles. For instance, the process of converting silver ions to silver nanoparticles using chitosan happens faster at elevated temperatures, which affects the size and crystallinity of the nanoparticles. In addition, elevated temperatures can enhance the oxidation of the functional groups in chitosan, thereby promoting the reduction of metal ions and resulting in the creation of carbonyl groups on the surface of the nanoparticles. Optimizing temperature is essential for regulating the rate and quality of nanoparticle formation.

### 5.4. Impact of Ionic Strength

The efficacy of CS adsorption can be influenced using the ionic strength of the reaction media. The existence of rival ions in the solution might hinder chitosan’s capacity to attach to the nanoparticles, thereby impacting the process of adsorption. Optimizing the interaction between CS and nanoparticles requires adjusting the ionic strength, which is crucial for successful modification and stability. The impact of ionic strength on the colloidal stability of CS–DNA NPs was investigated by Isadora et al. [99] through an examination of the interaction between CS of varying Mws (5, 10, 16, 29, 57, and 150 kDa) and calf thymus DNA. All deacetylated chitosans bind to DNA well at low pH values, and the diameters of the nanoparticles grow modestly in the ionic strength range of from 10 to 150 mM. The electrostatic repulsion between the charged CS chains adsorbed on the surface of the nanoparticles provides stability at low ionic strength. At ionic strengths of 150 and 500 mM, the stability of CS–DNA NPs declined significantly, leading to a commensurate drop in the thickness of the stabilizing shell. Only stable nanoparticles are formed when deacetylated chitosan interact with DNA with low ionic strength at pH 6.3.

### 5.5. Cross-Linking Agents

The selection and concentration of cross-linking agents play a crucial role when employing chemical coupling in the synthesis process. Utilizing cross-linking agents can improve the durability and physical characteristics of CS coating on the nanoparticles. The choice of suitable cross-linkers and their concentrations can impact the ultimate characteristics of the nanocomposites, including their longevity and functioning [100].

## 6. Uses for Chitosan-Based Nanocomposites

### 6.1. Purification of Water

The utilization of artificial pigments is progressively rising in industrial sectors, namely within the textile industry, resulting in significant water contamination due to their unprocessed release into aquatic environments. The textile industries utilize more than 10,000 distinct colorants, including pigments and dyes. Additionally, a staggering 7 × 10^5^ tons of synthetic dyes are generated globally each year [101,102,103]. A significant proportion of synthetic dyestuffs are released into the ecosystem without undergoing proper treatment, leading to the emergence of worldwide environmental issues [102]. The unreactive nature of dyes and their minute presence make their removal from water bodies a challenging undertaking. Due to its high concentration of reactive hydroxyl (-OH) and amino (-NH_2_) groups, along with the low cost of the biopolymer, chitosan is being explored for potential applications in water filtration. The electrostatic interaction between the protonated amino groups and the negatively charged dye ions facilitates the adsorption of acidic dyes using chitosan and modified CS [104]. Shen et al. [105] demonstrated that chelating interactions, as opposed to electrostatic interactions, are responsible for the removal of dyes from alkaline effluent [105].

Nanocomposites of CS have successfully removed dye from wastewater through many methods, including physical adsorption, ion exchange, hydrogen bonds, chemical bonding, and hydrophobic attractions. Ali et al. [106] synthesized CS-based composite fibers (MNPs/ZnPc–CS) and pellets containing zinc phthalocyanine (ZnPc)-supported metallic and bimetallic nanoparticles to absorb metal ions. The MNP/ZnPc–CS fibers were utilized as dip catalysts to reduce nitrophenols and azo dyes such as congo red and methyl orange. The results demonstrated that the produced composites had exceptional catalytic efficacy and reusability in the reduction of these dyes. The findings of several types of research about the elimination of colors from wastewater using nanocomposites made from chitosan are compiled in Table 2.

**Figure 7:**
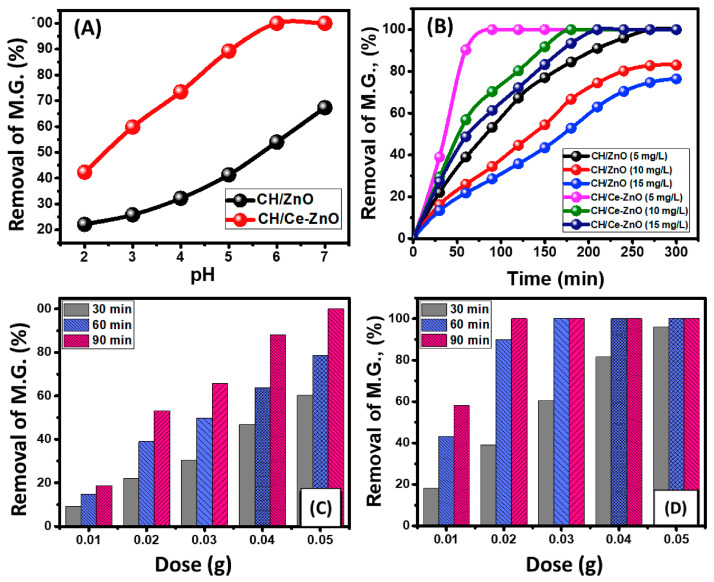
(**A**) Effect of malachite green degradation rates by CS/ZnO and CS/Ce–ZnO compounds as photocatalysts by pH value. (**B**) Study of the photodegradation of malachite green by CS/ZnO and CS/Ce–ZnO under visible light for different time periods and different initial concentrations. (**C**,**D**) Examination of the degradation results of Mg for three different time periods to determine the effect of CS/ZnO and CS/Ce–ZnO masses (reproduced with permission from Alaa et al. [107]).

In the study of Alaa et al. [107], the effect of pH on the photodegradation of malachite green dye by CS/ZnO and CS/Ce–ZnO was studied, as shown in Figure 7. The degradation rates of magnesium showed a continuous improvement in values with the gradual increase in the pH of the solution from pH 2 to about pH 7. The pH values detected for the best degradation of 5 mg/L of magnesium by CS/ZnO and CS/Ce–ZnO were pH 7 (67.3%) and pH 6 (100%), respectively.

**Table 2:** Nanocomposites based on chitosan for wastewater dye removal.

Nanocomposite Based on Chitosan	Metal/Dye	pH	Extraction Method	Ref.
CS/MoO_3_/TiO_2_	Methyl orange	No data	Degradation of photocatalysis under solar light	[108]
CS/Ag_3_PO_4_/CdS	Methyl orange	3–8	Catalytic photo-decolorization	[109]
CS/AgNPs	Methyl orange	3–11	Photocatalytic decolourization	[72]
CS/TiO_2_	Congo red, Rhodamine-B	3–11	Photocatalytic degradation in the presence of visible light	[110]
Palladium/CS	4-Nitrophenol	No data	Catalytic hydrogenation	[111]
CS/PVA/ZnO	Acid Black-1	No data	Adsorption	[112]
Bio-silica/CS	Acid Red 88	1–12	Adsorption	[113]
CS/AuNPs	4-Nitrophenol	No data	Catalytic reduction	[114]
CS/lignin/titania	Brilliant Black	No data	Adsorption	[115]

### 6.2. Metal Removal Using Metal Nanoparticles from Chitosan

In order to remove metals from wastewater during the wastewater treatment process, chitosan metallic nanoparticles are used in the adsorption batch procedure. Creating metallic nanoparticles based on chitosan by in situ or ex situ technologies is the first stage of this process. Then, using the solution casting method, these nanoparticle solutions are utilized to create films. Plastic or Teflon dishes or containers are used to dehydrate the films. Next, these films are cut exactly to the desired diameters and then submerged in different metal stock solutions of different concentrations [116]. Ideal conditions are used to evaluate the effects of CS and metal concentration on adsorption.

The removal of Cr (IV) was accomplished by adsorption by magnetic nanoparticles based on CS. The influence of pH (2–8) and temperatures (25 °C, 45 °C, and 65 °C) was examined by Ana Claudia et al. [117]. The maximum absorption was found to occur at pH 2, where the absorbed amount was 325 mg/g, and decreased to 125 mg/g when the pH was increased to 8.0. The absorption of chromium hexahydrate on the sorbent decreased from 295 mg/g at 25 °C to 209 mg/g at 65 °C. Research has typically indicated that increased concentrations of biopolymers and chitosan result in better heavy metal adsorption [118]. Nd^3+^, Dy^3+^, and Er^3+^ ion extraction was carried out using Fe_3_O_4_-C18-chitosan-DETA particles at 25 °C and a mean pH of 7. The surface deposition-stepwise grafting technique was used to successfully create the nanoparticles [119]. The order of the adsorption reaction was determined using kinetics data, revealing a pseudo-2-order process. Additionally, the adsorption isotherms were well-matched using the Langmuir equation. A comparative search was carried out to investigate the influences of CS on metallic nanoparticles for the adsorption of Cd (II) and Pb (II). Outstanding results were attained. Maximum removals of 79.24 mg/g of Pb (II) and 36.42 mg/g of Cd (II) were achieved by the Fe_3_O_4_/CSNPs. A simple one-step in situ co-precipitation technique was used to create the magnetic CS NPs [119]. Magnetic particles stabilized by chitosan are commonly used. To remove Cu (II) ions, Meng et al. [120] used superparamagnetic nanoparticles with a magnetic moment of 36 emu/g and a size range of from 8 to 14 nm. The Langmuir isotherm model was employed, resulting in a maximum adsorption capacity of 35.5 mg/g. Two chitosan derivatives were synthesized: one by crosslinking using glutaraldehyde and the other through functionalization using crosslinking and magnetic nanoparticles. The second derivative exhibited superior adsorption ability compared to the absence of magnetic nanoparticles [121]. CS-modified Mn ferrite NPs were produced using a microwave-assisted hydrothermal processing technique to eliminate Cu^2+^ ions from a water-based solution. The adsorption efficiency was determined to be 100% and 96.7% after 500 min at a pH of 6.5, using initial concentrations of Cu^2+^ ions of 100 and 50 mg/L. The Langmuir isotherm models were determined to be suitable for fitting the adsorption data. The Langmuir adsorption equilibrium constant, maximum adsorption capacity, and rate constants were derived based on the adsorption data. The results were consistent with the pseudo-2-order model proposed by Meng et al. [120] (Table 3).

**Figure 8:**
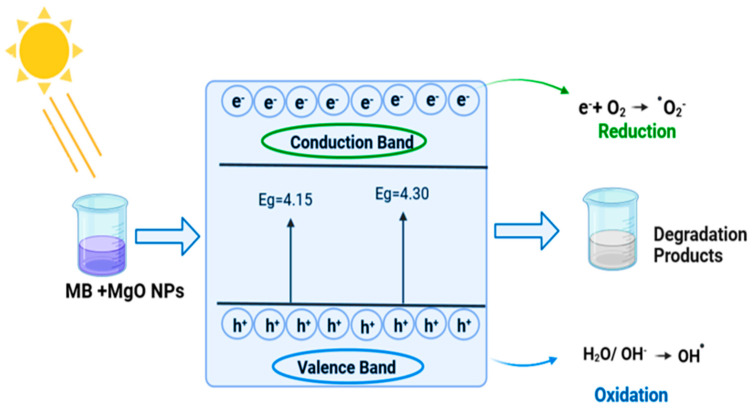
Photocatalytic degradation mechanism of the surface of MgONPs prepared by chitosan (reproduced with permission from Amor et al. [122]).

Another study [122] showed that MgONPs made from CS extracted from shrimp shells had superior photocatalytic activity and smaller band gap values than nanoparticles made from CS generated from crab shells. After 120 min of exposure, MgONPs from shrimp and crab shells, respectively, showed photocatalytic degradation efficiencies of 89% and 77%. The photocatalytic mechanism of methylene blue (MB) breakdown is shown in Figure 8. The first step in the process is the exposure of MgONPs made with chitosan to light. This excites the electrons in the valence band, which then leave behind positively charged holes in the band as they absorb energy and travel to the conduction band. By interacting with oxygen molecules (O_2_) in the solution, excited electrons in the conduction band create negatively charged superoxide radicals (•O_2_^−^), which aid in the pollutant’s breakdown. Concurrently, the valence band’s positive holes react with hydroxide ions (OH^−^) or water (H_3_O^+^), producing powerful oxidizing agents called hydroxyl radicals (•OH). By attacking the polluting chemicals, these radicals convert them into innocuous breakdown products. Under sunlight, the pollutant, such as MB, is efficiently degraded by the combined action of hydroxyl radical oxidation and superoxide radical reduction, demonstrating the photocatalytic properties of MgONPs.

**Table 3:** Utilization of CS metallic NPs for metal removal.

Chitosan/Metal	Metal Removal	Ref.
MnFe_2_O_4_–Chitosan	Adsorption of Cu^2+^	[92]
Chitosan–Fe_3_O_4_	Pb(II)	[123]
Chitosan–FeS	Cu(II)	[124]
Chitosan–Fe	Rare-earth metal ions	[119]
Fe_2_O_3_–Chitosan	Thorium (IV)	[108]
PEI@AC@Fe_3_O_4_–CS/PVA	Cr(VI)	[125]
Fe_3_O_4_–Chitosan	Cobalt and nickel	[126]
Chitosan–TiO_2_	Cr(VI)	[127]

### 6.3. Application of Photoacoustic Therapy

PAI, or photoacoustic imaging, is a cutting-edge technique in biomedical imaging that combines the advantages of high optical resolution and deep acoustic penetration. This technology has been extensively documented in the scientific literature [128,129]. PAI has the potential to provide detailed anatomical and functional information in various areas, such as breast cancer detection, measuring epidermal melanin, imaging brain structure and function, monitoring blood oxygenation, and estimating quantitative blood flow. This makes PAI a promising tool for both preclinical and clinical applications [129]. Exogenous contrast compounds are commonly employed to amplify the signals from PAI. Recent investigations have shown that chitosan–metal nanoparticles can serve as effective exogenous contrast agents for PAI. This is illustrated by the in vitro test results of chitosan-coated AuNS and chitosan-coated PdNPs, which were produced using a green technique. These results demonstrated a robust photoacoustic signal [129]. ThiTuong et al. [39] used the PAI system designed by them to obtain PA images of AuNS manufactured by chitosan. The setup of the PAI system was presented. The tubes containing AuNS exhibited strong signals that increased in magnitude as the AuNS concentration increased. In contrast, the control tube with phosphate-buffered saline (PBS) did not produce any signals. Therefore, AuNS could be a promising agent for enhancing the quality of PA imaging of tumors. By increasing the concentration of metal nanoparticles coated with CS, a stronger photoacoustic signal was detected. The findings indicate that light-absorbing chitosan NPs have the potential to be suitable contenders for PAI.

### 6.4. Application of Photothermal Therapy

Photothermal therapy (PTT) is a relatively new form of therapy that employs light to identify and treat medical issues [130]. This method has several advantages: minimal invasiveness, quick recovery, maintenance of natural tissue integrity, and a low rate of patient complications. CS–AuNPs have proven to be effective photothermal agents, as demonstrated by several research groups [131,132]. Breast cancer cells were used to assess the photothermal performance of CS-coated AuNS, which were produced using a green method [39]. The synthesized CS–Au nanostars demonstrated strong near-infrared (NIR) absorption. Upon exposure to NIR, the temperature of the AuNS solution coated with CS at a concentration of 60 μg/mL rose to 48.5 °C after 5 min, while an increase of about 4 °C was obtained for phosphate-buffered saline, and it demonstrated outstanding photothermal stability and excellent biocompatibility with both MDA-MB-231 and MG-63 cells. The combination of NIR laser and CS–Au nanostars successfully eliminated MDA-MB-231 breast cancer cells, proving their expertise in PTT. Our group conducted another study that examined the photothermal performance of CS–PdNPs. These nanoparticles were also synthesized using an environmentally friendly approach [38]. The CS–PdNPs successfully eradicated the MDA-MB-231 cancer cells through the conversion of photons into heat, demonstrating the nanoparticles’ potential for PTT [38].

The spatial arrangement of CS–metal NPs within the tumor determines the efficacy of PTT [133]. Hence, it is imperative to create CS–metal nanoparticles that specifically target cancer cells to ensure the efficient dispersion of nanoparticles within the tumor. Furthermore, PTT has the potential to be used in conjunction with other therapeutic modalities, such as immunotherapy, chemotherapy, radiation, or surgery, to optimize the efficacy of cancer treatment [134].

**Figure 9:**
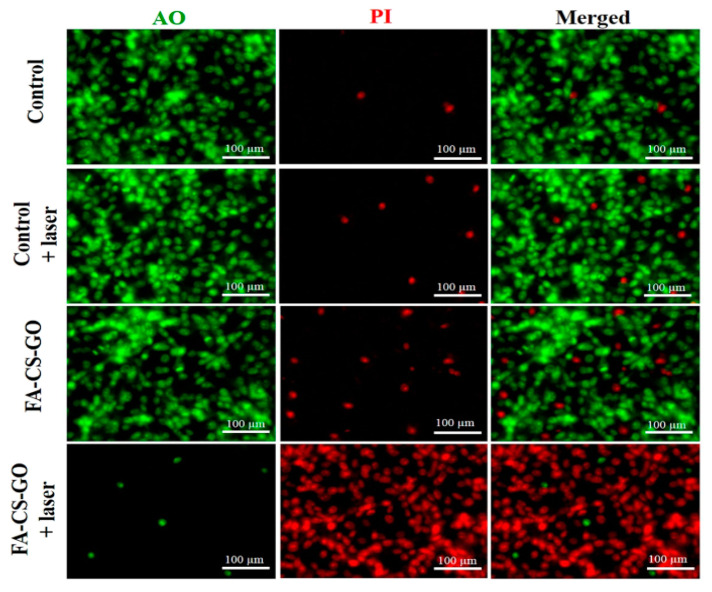
Fluorescence pictures of MDA-MB-231 cells co-stained with AO and PI following PTT treatments (reproduced with permission from Seung et al. [135]).

In a study by Seung et al. [135], functionalized folic acid-conjugated chitosan-functionalized graphene oxide (FA-CS-GO) was developed as a new type of multifunctional nanomaterial for near-infrared photoacoustic imaging-guided photothermal therapy (PTT) of cancer. The temperature of the FA-CS-GO solution (350 µg/mL) under the laser irradiation conditions readily reached 38.3 °C, 44.9 °C, 49.0 °C, and 51.8 °C, within 5 min of laser irradiation, with an output power of 0.5, 1.0, 1.5, and 2.0 W/cm^2^, respectively (Figure 9). Additionally, the in vitro results showed that FA-CS-GO was able to destroy cancer cells by 87% under laser irradiation, indicating that it can serve as an effective agent for PTT in vitro.

### 6.5. Application of Antibacterial Therapy

The problem of multidrug resistance is a significant challenge in modern medicine. To address this issue, scientists must prioritize the research and development of innovative and effective bactericidal materials. Due to their strong electrostatic interactions with lipopolysaccharides in the outer membranes of Gram-negative bacteria, CS–metal nanoparticles, which possess strongly positive charges, show significant potential as sensitive nanosensors and are cost-effective. For instance, it has been demonstrated that CS–AuNPs are highly efficient in killing bacteria [136]. The experimental results demonstrated the suppression of the typical proliferation of highly resilient bacterial strains. The authors also presented evidence indicating that the charge density of CS determined its antibacterial effectiveness. This feature is characterized by the presence of robust electrostatic contacts between CS and the charged surface of the lipid bilayer found in bacterial cell membranes. This suggests that the action mechanism of CS–AuNPs is non-specific in nature. The Au–CS nanocomposites produced by Mendoza et al. [137] exhibited antibacterial properties against *Escherichia coli* (Gram-negative) and *Staphylococcus aureus* (Gram-positive) bacteria, with the level of activity depending on the concentration. Based on the results from flow cytometry and SEM tests, the bacterial death mechanism induced by Au–CS colloids appears to involve cell wall breakdown and subsequent release of intracellular material. AgNPs are well-recognized as highly efficient antibacterial nanoparticles. Nevertheless, their potency is significantly elevated, but their practical use as an antibacterial agent is restricted. Chitosan can function as a nanocarrier and as a co-antibacterial agent in the presence of AgNPs. Sharma et al. [138] synthesized Ag–CS NPs and evaluated their antibacterial efficacy. The TEM images demonstrated the robust adhesion of the nanocomposite to the bacteria as a result of their elevated surface area and reactivity. Bacterial cells were subjected to treatment with the minimum inhibitory concentration (MIC) of Ag–CS NPs and with CS NPs at a concentration equivalent to CS concentration at the MIC of Ag–CS NPs (189.12 μg/mL) for several durations (30 min, 1 h, 3 h, and 6 h). The proportion of injured cells rose dramatically to 28.18% for MIC of Ag–CS NPs, in contrast to 14.97% for bacteria treated with CS NPs at 6 h. Positively charged nanoparticles are known to interact with bacteria, resulting in membrane disruption, intracellular component leakage, and, ultimately, cell death. AgNPs are known to penetrate cell wall barriers, resulting in poor permeability; hence, Ag–CS NPs exhibited enhanced antibacterial activity compared to bare CS NPs. Nanoparticles were anticipated to engage more intensely with the bacterial surface owing to their elevated surface area and reactivity, resulting in the loss of membrane integrity.

Appu et al. [28] show that a Cu–CS NP compound demonstrated bacteriostatic effects against both gram-negative and gram-positive bacteria. However, the activity had a greater effect on gram-negative bacteria, the variation in the bacterial cell wall structure between gram-negative organisms (with a single peptidoglycan layer) and gram-positive organisms (with many peptidoglycan layers) might be the cause of this discrepancy. The attachment of nanoparticles to the surface of a bacterium modifies its membrane characteristics, ultimately leading to its demise. In another study [41], ZnO NPs created with chitosan from crab shells showed a greater antibacterial effect on Gram-positive bacteria *Bacillus subtiliis* (20 ± 0.2 mm) than on gram-negative bacteria *Pseudomonas aeruginosa* (15 ± 0.25 mm).

### 6.6. Application of Photodynamic Therapy

PDT, or photodynamic therapy, is a treatment that utilizes low-intensity visible light and a photosensitizer (PS) to treat patients. When the photosensitizer is activated using visible light in the presence of oxygen, it generates cytotoxic substances that have the ability to eliminate or harm tumor cells. CS–metal NPs can serve as carriers for the photosensitizer in photodynamic therapy (PDT) when applied to the CS layer.

Chemical bonding between CS and the photosensitizer is possible. CS has functional groups that facilitate this process. For example, Hari et al. [139] used glutathione to create a multifunctional nanoparticle by binding Acridine orange to the surface of AuNPs manufactured using CS. Specifically, this nanoparticle enhances both PTT and PDT by targeting breast cancer cells. The experimental results demonstrated that AuNPs raised the temperature 45 °C within 2 min of irradiation time, and then it gradually increased to around 50 °C after 4 min of exposure. This confirms that there was greater photostability and a twofold increase in fluorescence intensity compared to free AO. Additionally, there was quicker absorption by cells. The present study demonstrates that incorporating photosensitizers into CS–metal nanoparticles enhances the efficiency of PDT and PTT.

PDT’s low tissue penetration makes it less effective in treating deep cancer tissue [140]. Therefore, to increase the efficacy of therapy at deeper depths, photosensitizers with the capacity to absorb near-infrared (NIR) light must be developed. Furthermore, the proper dispersion of photosensitizers using chitosan–metal nanoparticles may improve photodynamic therapy’s effectiveness. To improve the properties associated with medication action as well as the specificity of the target, CS–metal nanoparticles can be connected with targeting agents, such as antibodies or DNA/peptide-based linkers [141].

## 7. Conclusions

Chitosan has proven to be an incredibly versatile and effective multifunctional agent in the production and use of metal nanoparticles. Its unique abilities as a size-controllable agent, shape-directing agent, stabilizing agent, and green-reducing agent have facilitated the preparation of a variety of metallic nanoparticles and nanocomposites. Incorporating chitosan into the synthesis of nanoparticles not only improves the functionalization of these materials but also promotes sustainable and eco-friendly methods. Chitosan-based nanocomposites show great promise for several vital applications, such as the removal of metals from water and photoacoustic, photothermal, antibacterial, and photodynamic therapies, among other therapeutic modalities. The wide range of uses for chitosan highlights its important contribution to the development of nanotechnology and its applications, providing exciting new directions for future study and research in the environmental and biological domains.
